# Wellbeing, mental health knowledge and caregiving experiences of siblings of people with psychosis, compared to their peers and parents: an exploratory study

**DOI:** 10.1007/s00127-016-1222-7

**Published:** 2016-04-28

**Authors:** Jacqueline Sin, Trevor Murrells, Debbie Spain, Ian Norman, Claire Henderson

**Affiliations:** 1 Health Service and Population Research Department, David Goldberg Centre, Institute of Psychiatry, Psychology and Neuroscience, King’s College London, 16 de Crespigny Park, London, SE5 8AF UK; 2 Population Health Research Institute, St George’s, University of London, London, UK; 3 Florence Nightingale Faculty of Nursing and Midwifery, King’s College London, London, UK; 4 MRC Social, Genetic and Developmental Psychiatry Centre, Institute of Psychiatry, Psychology and Neuroscience, King’s College London, London, UK

**Keywords:** Psychosis/schizophrenia, Siblings/brothers/sisters, Family carers, Informal caregiving, Wellbeing

## Abstract

**Purpose:**

The wellbeing and caregiving experiences of family carers supporting people with psychosis has garnered increasing interest. Evidence indicates that the burden of caregiving can adversely impact on parents’ wellbeing, few studies have investigated whether this is also the case for siblings, who often take on caregiving responsibilities. This exploratory study investigated the wellbeing, mental health knowledge, and appraisals of caregiving in siblings of individuals with psychosis.

**Method:**

Using a cross-sectional design, 90 siblings completed three validated questionnaires: Warwick–Edinburgh Mental Wellbeing Scale (WEMWBS), Mental Health Knowledge Schedule (MAKS), and Experience of Caregiving Inventory (ECI). Data obtained were compared to general population norms and parent-carers’ scores. Multi-variable regression analyses were conducted to examine relationships between questionnaire scores and demographic characteristics including age, sex, birth order, marital status, accommodation and educational level.

**Results:**

Siblings, especially sisters, had significantly poorer mental wellbeing, compared to normative scores. Conversely, they had better mental health knowledge. Siblings and parent-carers had comparable high levels of negative appraisals of caregiving experiences, but siblings reported more satisfaction with personal experiences and relationships. Education level was a significant predictor for better mental health knowledge; there were no other relationships between siblings’ demographic factors and outcomes.

**Conclusion:**

Study findings suggest that siblings have overlapping as well as distinct needs, compared to parent-carers. Further research is required to better understand siblings’ experiences so as to inform development of targeted interventions that enhance wellbeing and caregiving capacity.

## Introduction

Psychosis is the most common severe mental illness, affecting approximately 1 % of the population [1, 2]. The onset of psychosis often peaks during late adolescence, leading to significant impairments, and potentially prolonged need for treatment. It is widely recognised that coping with psychosis can prove challenging, not just for the individual themselves, but for everyone in their familial network [1–4]. Importantly, empirical data indicate that individuals who receive support from family members have a better prognosis and improved quality of life [1, 5–8]. However, the burden of caring can incur clinically significant levels of distress, depression, and anxiety: mental health of carers is inversely correlated with the amount of care they provide [3, 4, 8]. Also, the wellbeing of carers is associated with their caregiving capacity, that is, poorer wellbeing affects propensity to provide adequate support [6, 8]. Consequently, several studies have examined carer-specific interventions, which aim to enhance knowledge and understanding of psychosis, capacity for coping and coping strategies, to improve sense of self-efficacy [9–12]. Appraisals of caregiving experiences are crucial for determining carers’ wellbeing [8, 13–15].

While traditionally parents have assumed the role of named carer, usually mothers [6–12], there is increasing awareness that siblings also take on caregiving roles [16–18], but few studies have investigated their needs and experiences. Also, most studies have recruited siblings who are in their 40s and 50s, and after they have taken on the key caring role from their parents [19–26]. A recurring theme is that siblings perceive themselves to be under pressure to become carers, and they experience a subjective sense of burden. Also, siblings experience a range of psychological and socioeconomic stressors, not dissimilar to those reported by parent-carers [17, 26–28]. These include: shock and confusion when psychotic symptoms initially manifest; grief and a sense of loss; distress; difficulties with coping; and stigma associated with mental illness. Additionally, there are several sibling-specific worries, such as “survival guilt” and concerns about heredity and genetic risk factors [16–18, 27, 28]. In addition to the well-established negative correlation between being a family carer and poorer wellbeing [3, 4, 8], several other significant risk factors have been identified for this outcome. These include: being female, aged between 45 and 54, not in a stable relationship, and without a degree level qualification [29, 30]. Furthermore, studies focusing on individuals with psychosis indicate that there is an association between family carers’ poor wellbeing and short duration of illness [14, 15]. Family carers of individuals with recent onset psychosis report higher levels of subjective and objective burden and distress, compared to individuals who have been carers for a longer time [6]. In the first quantitative study exploring the quality of life (QoL) of siblings of young people experiencing first episode psychosis (FEP), female and younger siblings living with their unwell brother/sister had lower QoL [31].

### E Sibling Project

The E Sibling Project (http://siblingpsychosis.org, ISRCTN0116694) was the first study to evaluate an internet-based psychoeducational intervention for siblings of individuals experiencing a FEP [32] who may have high levels of burden and distress [14–17, 33, 34]. This paper examines the mental health knowledge, wellbeing, and appraisals of caregiving experiences, through analysing baseline data of participants recruited to the E Sibling Project (*n* = 90). The current study was exploratory in nature and had three aims: (1) to compare knowledge and wellbeing of siblings with those reported for age-matched individuals in the general population; (2) to compare FEP siblings’ appraisals of caregiving experiences with those of parents; and (3) to establish whether siblings’ knowledge, wellbeing and appraisals of caregiving experiences differed according to demographic characteristics known to increase risk for reduced QoL and wellbeing, namely: sex, age, birth order, marital status, accommodation, and education level [29–31].

## Methods

### Design

This was a cross-sectional correlational study which made comparisons with external data sources and explored associations between measures of wellbeing, mental health knowledge and appraisals of caregiving experiences and siblings’ characteristics prior to randomisation to the E Sibling Project. Published English population survey statistics (Health Survey for England (HSE) [29, 30] and Attitude to Mental Illness Survey (AMIS) [35, 36]) were used to provide population norms for wellbeing and mental health-related knowledge outcomes. FEP parent-carers’ data about caregiving appraisals were obtained from a prior study (*n* = 68, 87 % were parents) [37].

### Participants

Two cohorts of sibling participants were recruited to the E Sibling Project between 2013 and 2015. An initial cohort was recruited via non-governmental organisations (NGOs) for a feasibility study [38]. A second cohort was recruited to the randomised controlled trial (RCT), from 26 Early Intervention in Psychosis Services (EIPS) in England [32], providing multi-disciplinary team input to people aged between 18 and 35, experiencing FEP [1, 39].

We included siblings, aged 16 or over, who were either biologically related, step- or half-siblings, or related through adoption. Siblings were required to have at least weekly contact, but they did not need to live together. Participants had to be fluent in English, and have access to the online intervention [32, 38].

### Ethical approval

Ethical approvals were obtained by the NHS Research Ethics Committee (REC reference: 12/LO/1537), and Research and Development departments at participating health trusts. Siblings could self-refer, or receive information from clinicians or researchers. Of note, ethical approvals were in place to recruit siblings without the need for consent from service users, nor were personal data about service users obtained.

### Measures

The Warwick–Edinburgh Mental Wellbeing Scale (WEMWBS) is a self-report measure of positive mental wellbeing that comprises 14 positively worded statements, rated on a 5-point Likert scale [40]. Possible scores range from 14 (minimum) to 70 (maximum); the higher the score the better the individual’s mental wellbeing. WEMWBS has been widely used in epidemiological studies, including the Health Surveys in England and Scotland [29, 30, 41].

Siblings’ knowledge of mental health was assessed using the Mental Health Knowledge Schedule (MAKS) [42]. MAKS has two sections: the first section has six questions to investigate participants’ knowledge of mental health; the second section has six further questions for establishing levels of recognition and familiarity with various conditions and also to help contextualise the responses to other items. Possible score ranges from six (minimum) to 30 (maximum). The higher the score, the better level of knowledge of mental health [42]. MAKS has been widely used in large scale studies, notably the evaluation of Time To Change [43].

Siblings’ experiences of caregiving was assessed using the Experience of Caregiving Inventory (ECI) [3] which comprises 66 items with ten subscales describing: difficult behaviours; negative symptoms; stigma; problems with services; effects on family; need to backup; dependency; loss; positive personal experiences and good aspects of relationship. Each of the 66 items contains a brief statement of experiences of caring and participants rate each item on a 5-point ordinal scale, in the last one month. Negative appraisal is the sum of the eight negative subscales (possible scores range from 0 to 208; higher scores indicate poorer negative appraisal) and positive appraisal is the sum of the two positive subscales (possible scores range from 0 to 56; higher scores indicate better positive appraisal) [3, 44, 45].

### Analysis

Data handling and editing were undertaken using SPSS software version 22 (IBM SPSS Inc., Chicago IL). Descriptive statistics for demographic and outcome variables were computed. For continuous outcome measures, means, standard deviations (SD) and 95 % confidence intervals (95 % CI) were calculated to represent the sample norms. Siblings’ mental wellbeing and mental health knowledge were compared to age-matched population norms on the WEMWBS (i.e. HSE-2013 [29, 30]) and on the MAKS (i.e. AMIS-2014 [35, 36]). The ECI positive and negative subtotals of the FEP sibling sample were compared to a sample of FEP parent-carers [37].

Independent-samples t tests, one-way analysis of variance (ANOVA) and correlation analyses using eta-squared (*η*^2^) were conducted to determine whether a priori identified siblings’ demographic variables (i.e. age, sex, birth order, accommodation—living together or not, marital status, and education level—having a degree level qualification or not) were associated with each of the outcome variables (i.e. wellbeing, knowledge, positive and negative appraisals of caregiving experiences) within the sibling sample. To determine the strength of the relationships, results were interpreted according to Cohen’s guidelines [46]. Given the exploratory nature of the research, alpha was set at 0.05 for all analyses. No adjustments were made for multiple comparisons, as they can result in higher type II errors, reduced power, and increased likelihood of missing significant findings [47]. Furthermore, multi-variable regression analyses were undertaken to establish how much of the variance in siblings’ clinical outcomes can be explained by their characteristic variables.

## Results

Twenty siblings consented to participate in the feasibility study, 19 of whom (95 %) completed baseline measures [38]. Of 104 siblings who consented to take part in the RCT, 71 (68 %) completed baseline outcome measures [32]. This resulted in a total sample of 90 siblings (73 % of those initially consented) whose data are described in this paper.

### Demographic characteristics of siblings

Table 1 summarises participants’ demographic characteristics. Most participants were female (85 %). Younger sisters (37 %) and older sisters (48 %) out-numbered younger brothers (8 %) and older brother (7 %). Approximately one-third of participants lived with their unwell sibling (and often with their parents). Less than half of all respondents were in a stable relationship (47 %) and just over half described themselves as single (53 %). Just over half (52 %) were educated to degree level or above, and the rest (48 %) had achieved secondary school or trade training qualifications.

**Table 1:** Summary of socio-demographic characteristics of siblings

Characteristics	Feasibility study sample (*n* = 19)	RCT sample (*n* = 71)	Total sample (*n* = 90)
Age in years, mean (SD)	35.42 (9.58)	25.41 (6.69)	27.52 (8.41)
Range	20–58	16–53	16–58
Sex			
Female, *n* (%)	16 (84.2)	61 (85.9)	77 (85.1)
Male, *n* (%)	3 (15.8)	10 (14.1)	13 (14.4)
Marital status, *n* (%)			
Single	10 (52.6)	38 (53.5)	48 (53.3)
Married or cohabiting	9 (47.4)	33 (46.5)	42 (46.7)
Ethnicity, *n* (%)			
Caucasian	15 (78.9)	45 (63.4)	60 (66.6)
Black	1 (5.3)	10 (14.1)	11 (12.2)
Asian		6 (8.5)	6 (6.7)
Mixed race	3 (15.8)	10 (14.1)	13 (14.4)
Vocational status, *n* (%)			
Full/part time education	3 (15.8)	30 (42.3)	33 (36.7)
Full/part time work	14 (73.7)	34 (47.9)	48 (53.3)
Other, e.g. retired, unemployed	2 (10.5)	7 (9.9)	9 (10)
Birth order, *n* (%)			
Younger sister	5 (26.3)	29 (40.8)	34 (37.8)
Younger brother	1 (5.3)	6 (8.5)	7 (7.8)
Older brother	2 (10.5)	4 (5.6)	6 (6.7)
Older sister	11 (57.9)	32 (45.1)	43 (47.8)
Education level, *n* (%)			
Completed secondary school or trade training	1 (5.2)	36 (50.7)	37 (41.1)
Completed a tertiary degree or beyond	18 (94.8)	35 (49.3)	53 (58.9)
Accommodation, *n* (%)			
Living with unwell sibling	1 (5.3)	26 (36.6)	27 (30)
Not living with unwell sibling	18 (94.7)	45 (63.4)	63 (70)
Unwell siblings’ characteristics	*N* = 18^a^	*N* = 71	*N* = 89
Age in years, range, mean (SD)	20–52, 33.05 (8.48)	15–57, 24.7 (6.74)	15–57, 26.46 (7.9)
Female, *n* (%)	7 (38.9)	27 (38.1)	34 (37.8)
Male, *n* (%)	11 (61.1)	44 (61.9)	56 (62.2)
Length of time in treatment, in months, range, mean (SD)	No information	*N* = 71 1–79, 21.8 (15.6)	
0–1 year, number (%)		25 (35.2)	
>1 to 2 years, number (%)		20 (28.2)	
>2 to 3 years, number (%)		17 (23.9)	
>3 but <5 years, number (%)		9 (12.7)	

### Clinical outcomes of siblings

Participants’ clinical outcomes are summarised in Table 2, along with normative data for comparison samples.

**Table 2:** Summary of outcome measures of sibling samples and external data sources

Outcome measures	Feasibility study sample (*n* = 19)	RCT sample (*n* = 71)	Total sample (*n* = 90)	External data sources
WEMWBS				From HSE 2013
Range	39–64	16–65	16–65	*n* = 2746 general public, aged 16 to 54
Mean (SD)	50.67 (7.15)	45.97 (10.29)	46.81 (9.79)	51.86 (8.42)
MAKS				From AMIS 2014
Range	21–28	11–28	11–28	*n* = 1100 general public, aged 16–58
Mean (SD)	24.50 (2.28)	23.23 (2.99)	23.49 (2.89)	22.88 (3.36)
ECI negative subscale total				From Onwumere 2008
Range	52–160	13–168	13–168	*n* = 68 FEP parents, mean age (SD) = 47.1 (9.73)
Mean (SD)	96.87 (23.43)	101.44 (31.78)	101.38 (30.49)	100.7 (37.1)
ECI positive subscale total				From Onwumere 2008
Range	24–39	15–54	15–54	*n* = 68 FEP parents, mean age (SD) = 47.1 (9.73)
Mean (SD)	32.69 (5.06)	31.89 (8.96)	31.97 (8.23)	28.6 (9.5)

*WEMWBS* Warwick–Edinburgh Mental Wellbeing Scale, *MAKS* Mental Health Knowledge Schedule, *ECI* Experience of Caregiving Inventory, *HSE* Health Survey for England, *AMIS* Attitude to Mental Illness Survey, *Onwumere 2008* reference item 37

### Comparisons of siblings’ clinical outcomes with external data sources

#### Comparison of siblings’ and general population’s mental wellbeing

Of the total sample of 90 siblings with ages ranged from 16 to 58 years, their mean WEMWBS of 46.81 (SD 9.79) was significantly lower than the age-matched population norms of 51.86 (SD 8.42) (calculated from a sample of 2746 individuals aged 16–54, 44 % male from published data) [30]. A two-sample t test showed that the mean difference of −5.05 WEMWBS scores was statistically significant between our sibling sample and the HSE sample (*t* = −4.83, 95 % CI −7.10 to −3.00, *p* < 0.001). Mean WEMWBS of the 77 female siblings was 46.69 (SD 9.82), significantly lower than the HSE female population mean WEMWBS of 51.58 (SD 10.36) (*t* = −4.25, 95 % CI −7.14 to −2.64, *p* < 0.001). However, mean WEMWBS of the 13 male siblings (47.54, SD 10.05) was not significantly different from the HSE male population mean of 52.12 (SD 11.44) (*t* = −1.63, 95 % CI −10.08 to 0.92, *p* = 0.010). See Table 2.

#### Comparison of siblings’ and general population’s knowledge

The mean MAKS of siblings [*n* = 90, mean age (SD) = 27.52 (8.41)] was 23.49 (SD 2.89) and one-sample *t* test showed that our sample’s MAKS score was significantly higher (*t* = 1.995, 95 % CI 0.00 to 1.22, *p* = 0.049) than that of the age-matched AMIS mean MAKS score of 22.88 (SD 3.36) (based on 1100 individuals aged 16–58) [35]. See Table 2.

#### Comparison of FEP siblings’ and parents’ ECI

We compared the ECI positive and negative subtotals of siblings [*n* = 71, mean age (SD) = 25.41 (6.69)] whose brother or sister had a FEP (see Table 2) with those of a sample of FEP parent-carers [*n* = 68 (88 % female), mean age (SD) = 47.1 (9.73), mean ECI positive subscale total = 28.6, SD = 9.5; mean ECI negative subscale total = 100.7, SD = 37.1] [37]. A one-sample t test showed that the siblings had a significantly higher ECI positive subtotal scores than the parent-carers (*t* = 3.092, 95 % CI 1.17–5.41, *p* = 0.003) but no significant difference in the ECI negative subtotal scores between the two samples (*t* = 0.195, 95 % CI = −6.79–8.26, *p* = 0.846).

### Association between siblings’ demographic characteristics and clinical outcomes

Multi-variable regression analysis revealed that all demographic factors showed little, if any, association with the four clinical outcomes (see Table 3). In this sample, siblings’ age, sex, birth order, accommodation, marital status and education level, were not associated with their WEMWBS scores. Likewise, siblings’ demographic factors did not show any significant association with their positive and negative appraisals of caregiving experiences. While univariate analysis identified that older siblings, those who were in a stable relationship, or those educated to degree level or above have better mental health knowledge, only education level remained a significant predictor of a higher MAKS score in siblings in the multi-variable analysis when other demographic variables were controlled for (see Table 3).

**Table 3:** Multi-variable regression analyses with the four clinical outcomes serving as dependent variable in each model and the predictors including the six demographic variables set a priori

Predictors^a^	ECI positive subtotal				ECI negative subtotal				MAKS				WEMWBS			
	*B*	95 % CI	*p*	*η* ^2^	*Β*	95 % CI	*p*	*η* ^2^	*B*	95 % CI	*p*	*η* ^2^	*B*	95 % CI	*p*	*η* ^2^
Age	−0.013	−0.258 to 0.232	0.918	0.000	0.247	−0.659 to 1.153	0.589	0.004	0.007	−0.073 to 0.086	0.869	0.000	0.209	−0.80 to 0.499	0.154	0.024
Sex	−2.309	−7.317 to 2.698	0.362	0.10	6.247	−12.265 to 24.760	0.504	0.005	−0.720	−2.339 to 0.899	0.379	0.009	0.535	−5.387 to 6.456	0.858	0.000
Birth order	−0.549	−4.326 to 3.229	0.773	0.001	−6.055	−20.021 to 7.911	0.391	0.009	−0.908	−2.129 to 0.313	0.143	0.026	2.971	−1.496 to 7.439	0.189	0.021
Accommodation	−2.685	−7.230 to 1.859	0.243	0.016	−7.994	−23.795 to 8.806	0.347	0.011	−0.544	−2.014 to 0.925	0.463	0.007	0.847	−4.527 to 6.221	0.755	0.001
Marital status	0.559	−3.243 to 4.361	0.771	0.001	1.491	−12.565 to 15.547	0.833	0.001	−0.706	−1.935 to 0.523	0.256	0.015	1.724	−2.772 to 6.220	0.448	0.007
Education level	0.315	−4.010 to 4.641	0.885	0.000	9.724	−6.268 to 25.717	0.230	0.17	−1.911	−3.310 to 0.513	0.008	0.082	1.491	−3.625 to 6.606	0.564	0.004

*B* unstandardised regression coefficient, *95* *% CI* 95 % confident interval; *p* probability, *η* ^*2*^correlation coefficient eta-squared, *ECI* Experience of Caregiving Inventory, *MAKS* Mental Health Knowledge Schedule, *WEMWBS* Warwick-Edinburgh Mental Wellbeing Scale

^a^Excluding age, predictor variables were categorised in binary terms: sex—male or female; birth order—younger or older than the service user; accommodation—not living with the service user or living together with the service user; marital status—single or in a stable relationship; education level—below or above degree level qualification; the latter category of the predictors were used as the reference groups in the multi-variable regression

## Discussion

Our study findings indicate that siblings of individuals who have psychosis tend to have poorer mental wellbeing compared to the general population in England [30]. More specifically, sisters were found to fare worse than their same sex counterparts. Mental health knowledge, on the other hand, was found to be better than general population means [35, 36]. In terms of appraisals of caregiving experiences, siblings of individuals with FEP scored similarly on the ECI negative subtotals, but significantly higher on the ECI positive subtotals, compared with parent-carers, as their scores indicated that they viewed their experiences and relationship more positively [37]. Siblings’ demographic characteristics did not significantly predict clinical outcomes, with the exception of the relationship between being educated to at least degree level, and higher MAKS score. These findings suggest that siblings’ mental wellbeing and caregiving experiences are potentially similar to those reported for carers in the wider literature (e.g. [3, 4, 13, 30, 44, 45]). All siblings recruited to the E Sibling Project regarded themselves as being actively involved in providing support for their unwell brother/sister, and were likely to actively seek support for themselves. It is possible that the intensity of siblings’ involvement in caregiving activities may have overshadowed the categorisation of their demographic characteristics; that is, whether siblings live together may have little association with the amount of emotional support they provide.

As no published studies investigating carers’ mental wellbeing using the WEMWBS were found, no comparison could be drawn between siblings and other types of family carers. Given that physical and mental health morbidity rates are high in family carers [1–4], and that QoL is often adversely affected (in family members) during the onset of psychosis and associated risk factors, such as harm to self or others [6, 15, 17, 31], it is unsurprising that siblings’ wellbeing is compromised, in part, by the impact of psychosis. Siblings’ low WEMWBS scores may reflect the burden of caregiving, or disruption of existing familial support structure [16, 17, 48], as well as other vulnerabilities intrinsic to the wider family network, such as poverty, the need to care for multiple people, and consequent social problems [3, 4, 45]. Further investigation into the mental wellbeing of siblings is warranted given the strong relationship between WEMWBS scores and multiple psycho-socio-economic variables [29, 30, 41].

Our study results indicate that siblings generally have a better level of mental health knowledge, concurring with the findings of the Attitude to Mental Illness Survey [36]. This general population survey identified several demographic variables associated with a higher MAKS score, including: being female; higher socio-economic status; and knowing someone with a mental health problem [36, 43]. These characteristics were prevalent amongst our sibling sample which was composed of more sisters than brothers and a high proportion of individuals educated to degree level.

Our findings suggest that caregiving experiences may also be associated with positive appraisals in ‘well’ siblings of individuals affected by FEP; a finding which has been reported elsewhere, whereby supportive sibling relationships are beneficial for service users’ quality of life and prognosis [19, 20, 48, 49]. It is possible, that positive appraisal in this sample may be attributed to regular contact and proximity in ages between siblings [16, 17, 48, 49]. Also, with the background of growing up together in a shared cultural heritage, siblings may be particularly aware of service user’s social and emotional needs. Unlike parent-carers who typically have responsibility for practical caring demands (e.g. providing accommodation, financial support, housework), siblings are more likely to initiate and share social overtures, social opportunities and aspirations with their brother/sister (such as social outings, introduction to friends or education/work opportunities) [17, 48, 49]. Our findings support previous research which has identified that the illness experience, in some cases, bring the family closer together and enhance empathy toward other family members [17].

Contrary to our hypothesis which anticipated that siblings were likely to have lower ECI negative subtotal scores than parent-carers, our FEP sibling sample did not fare any better in negative caregiving experience. ECI negative subscales cover questions on the carer’s perception of a range of caregiving issues, including: difficult behaviours; stigma; problems with services; dependency; and loss [3]. Our findings may suggest that although parents are often the formally identified carers (i.e. by services) who make most contacts with social and health services to help engage their child with support and resources, siblings are also involved in such encounters and demands. A small body of research focusing on siblings has identified that they often play a substantial role in supporting their unwell brother/sister as well as their parents and extended family, over a prolonged duration [16–20, 25].

### Limitations

This study has several limitations. First, we acknowledge that 20 % (*n* = 19) of participants were recruited directly from NGOs, and they may represent a subgroup of siblings who are more likely to help-seek. The remainder of our sample (*n* = 71) were recruited through EIPS; these siblings were likely have had a good relationship with their unwell brother/sister, or were recognised as a carer by the services. Thus, it is possible that siblings who have less (or no) contact with services may not be represented within our sample. Second, as siblings’ participation in the study did not require consent from service users, we did not collect data on potential illness variables which may serve as confounds, such as service users’ symptomatology versus siblings’ appraisal of their difficult behaviour, or service users’ social functioning versus siblings’ perception of loss or dependency. Third, although the eligibility criteria stipulated that siblings using secondary or specialist mental health services themselves were not eligible for inclusion in the study, we did not perform any clinical screening to rule out undiagnosed or untreated mental health problems. Depressive or anxiety symptoms, for example, may have affected scores on outcome measures. Fourth, sisters out-numbered brothers in our sample, thereby limiting comparisons between sexes, sex and birth order. Finally, we compared FEP siblings’ ECI scores with an independent FEP parent sample. Comparisons between siblings’ with their own parents’ ECI scores would have had the advantage of minimising potential confounders in terms of symptomatology and other factors inherent in the family context, but was not feasible.

### Clinical and research implications

Further to the well-established research evidence on family carers’ increased morbidities due to the burden of caregiving, the findings of this study suggest that siblings of individuals affected by psychosis suffer poor mental wellbeing and negatively appraise their caregiving experiences. These findings suggest that siblings need support and access to services. This is important given the well-established positive relationship between carers’ wellbeing and their caregiving capacity [4, 6, 8]. Furthermore, recent research on FEP siblings by Bowman and colleagues [16, 31, 50] has shown that psychosis often brings negative impacts on the relationships between the well and unwell siblings. These findings, coupled with the known correlation between the better quality in siblings’ relationships (especially around their late teenage and early adulthood years) and the increased likelihood of siblings involving in their brother/sister’s care in the long run [19, 20], have implications for early and targeted interventions for this vulnerable group. Timely interventions to promote siblings’ wellbeing and appraisal of caregiving experience, may not only bring short term gains in their own clinical outcomes, but also ease their transition into the key caring roles in the future. Further research is needed to enhance, adapt and/or develop interventions which optimise flexible access and delivery to siblings as well as address their unique concerns in addition to generic carers’ needs.
